# Sex and adrenal hormone alteration in Ecuadorian adolescents with home and school proximity to floriculture crop area

**DOI:** 10.1186/s12940-026-01291-x

**Published:** 2026-04-25

**Authors:** Georgia L. Kayser, Jasen Zhang, Azarakhash Baghdad, Jenny Wei, Briana N.C. Chronister, Alhelí Calderón-Villarreal, Kimberly C. Brouwer, Jose Suarez-Torres, Franklin De La Cruz, Jose R. Suarez-Lopez

**Affiliations:** 1 University of California, San Diego, USA; 2 California State University San Marcos, San Marcos, USA; 3 Fundación Cimas del Ecuador EC, Quito, Ecuador

**Keywords:** Endocrine disruption, Pesticides, Children and adolescents, Hormone, Agriculture

## Abstract

**Background:**

Pesticides frequently used in agriculture, including organophosphates, glyphosate, and 2,4-D, have endocrine disrupting potential. We previously found that residential proximity to floriculture crops can increase the potential for pesticide exposure of children and adolescents. Little is known about whether residential or school proximity to crops can affect their hormone levels.

**Methods:**

We examined 507 adolescents (12–17 years, 51% female, ESPINA cohort) living in agricultural communities in Ecuador. Salivary dehydroepiandrosterone (DHEA), testosterone, estradiol, and cortisol were measured. Using linear regression, we analyzed the association of hormones with residential distance to floricultural crops and crop areas near homes and schools within various radii (150 m, 200 m, 300 m, and 500 m).

**Results:**

The median (25th-75th percentile) residential proximity to floricultural crops was 312.6 m (112.3, 653.2), and 49% of adolescent participants lived within 300 m. Among these, the median (25th-75th percentile) crop area within 300 m was 8000m2. Within 150 m of children’s homes, doubling crop area was associated with lower concentrations of testosterone in boys (-9.14% (95% CI: -15.08%, -2.79%)). Doubling crop area within 300 m of homes was associated with greater cortisol concentrations in boys (8.05% (95% CI: 0.45%, 16.23%)). Doubling crop area within 500 m of homes was associated with lower estradiol in boys (-4.82% (95% CI: -9.26%, -0.17%)). Doubling crop area within 100 m of children’s schools was associated with lower estradiol in boys (-6.70% (95% CI: -11.78%, -1.34%)).

**Conclusions:**

We found greater crop area near homes was associated with testosterone, estradiol, and cortisol alterations in boys but not girls. Greater crop area near schools was associated with estradiol alterations in boys. This research suggests home and school proximity to pesticide spray site pose a risk to the developing endocrine systems of children.

**Supplementary Information:**

The online version contains supplementary material available at 10.1186/s12940-026-01291-x.

## Highlights

1. Nearly half of adolescents lived within 300m of floricultural crops.
2. Doubling crop area within 150m of homes was linked with lower testosterone in boys.
3. Doubling crop area within 300m of homes was linked to higher cortisol levels in boys.
4. Doubling crop area within 500m of crops was associated with lower estradiol in boys.
5. Doubling crop area within 100m of schools was linked with lower estradiol in boys.

**Supplementary Information:**

The online version contains supplementary material available at 10.1186/s12940-026-01291-x.

## Introduction

People living in agricultural communities have increased risk of pesticide exposure through direct contact and off-target drift of pesticides from crops onto nearby homes [1–4]. Residential proximity to agricultural crops is associated with greater pesticide levels in household dust [1, 5] and higher urinary biomarkers of pesticides in children and adults [5–7]. In a study by Brouwer et al., [8], more than 50% of the total pesticides sprayed in an area was moved away by wind or evaporation, and this happened most prominently within the first 100 m, but it can be detected up to several kilometers away from the center of the sprayed area. These para-occupational pesticide exposures may not necessarily result in acute clinical toxicity, but chronic exposures may increase the risk of adverse health effects [9–13].

Certain classes of pesticides have endocrine disrupting properties and can cause hormonal imbalance through multiple mechanisms such as altering absorption, secretion, and metabolism of hormones [14–16]. Pesticide disturbances in steroidogenesis pathways result in hormonal imbalances and may lead to adverse effects such as changes in the onset of puberty and fertility problems [17]. Exposure to insecticides, including organophosphates (OP), carbamates, organochlorines, neonicotinoids and pyrethroids, are linked with alterations in the sex steroid hormones testosterone, estradiol, DHEA and cortisol among adults [18–25]. However, to our knowledge, this relationship has only been studied among adolescents in one recent study, by our group, of insecticide biomarkers and hormone alteration [26].

Puberty is driven by sex hormones, which trigger the peripheral signs of sexual maturation when released at greater concentrations [27]. Dehydroepiandrosterone (DHEA) influences body odor, pubic hair and prepubertal growth for both sexes. Testosterone strengthens muscles and bones, stimulates genital development, and sperm development in males, while in females it helps produce new blood cells and maintain bone health. For females, estradiol stimulates breast development [27]. Sex hormone levels increase as adolescents reach adulthood [28, 29]. For instance, testosterone is around 45 times higher by adulthood compared to prepubertal stages in male adolescents [30]. Estradiol increases from childhood into adulthood, and for females, once menstruating, concentration changes occur throughout the menstrual cycle, in childbirth and in menopause [31]. In an individuals’ life course, cortisol increases in adolescence, decreases in their 20 s and 30 s, stabilizes in their 40 s and 50 s, then rises again later in life [32]. DHEA also increases through puberty and into early adulthood, peaking in the 20 s, and then gradually declines over time. As a precursor to testosterone and estradiol [33], DHEA plays a foundational role in the hormonal changes of puberty and adulthood.

In both animal and human studies, exposure to insecticides—including organophosphates (OP), pyrethroids, and neonicotinoids, and some herbicides like atrazine and glyphosate, were largely negatively associated with testosterone. Animal studies, primarily in rats and mice, found lower testosterone levels in the groups chronically treated with a neonicotinoid (imidacloprid or acetamiprid) [34–39], an OP (chlorpyrifos) [40], pyrethroids (cypermethrin and deltamethrin) [41], or carbamates (methomyl) [42]. Several epidemiological studies in agricultural workers suggest inverse associations between testosterone levels and exposure to OP (diethyl phosphate, chlorpyrifos, ethyl parathion and methamidophos), or pyrethroid concentrations and reduced testosterone levels in men [19–21, 24, 43–45]. Additional evidence from the U.S. and China suggests that pyrethroid insecticides affect Leydig cell function which could lead to a decrease in testosterone production [46] and [19].

While most studies suggest an inverse association between exposure to OPs, pyrethroids, or neonicotinoids and testosterone, a cross-sectional study of 133 male Thai farmers and a longitudinal study of 143 Mexican farmers reported a positive association with OP metabolites [22] and found no significant associations with OP metabolites and serum levels of testosterone, respectively [47]. The mixed results may reflect small sample sizes, limiting the power to detect small differences in effect. Additionally, pesticide class and exposure levels are also important factors in epidemiological studies [48]. In a recent study of 522 Ecuadorian adolescents who grew up near agriculture spray sites, endocrine disruption for specific metabolites was observed, the OP urinary metabolite, malathion dicarboxylic acid, was positively associated with testosterone while para-nitrophenol, a non-specific metabolite that reflects exposures to the OP parathion, but also of dyes, explosives and certain pharmaceuticals like acetaminophen, was negatively associated with testosterone in boys [26]. Although there are fewer epidemiological studies of neonicotinoids, a study of 2014 male and female American participants in the National Health and Nutrition Examination Survey found total testosterone was 37.8% lower with a tenfold increase in total urinary neonicotinoids [25].

While lesser studied, research suggests exposure to various insecticides—OPs, pyrethroids, neonicotinoids, and carbamates— and some herbicides—atrazine—can disrupt estradiol. Limited research points to mixed results depending on the type of pesticide and metabolite studied. Animal studies in mice and fish exposed to pyrethroids or OPs, had higher and lower levels of estradiol [49–51]. Estradiol had a marginally significant positive trend with OP metabolites (diethylphosphate and diethylthiophosphate) in a longitudinal study of 136 male floriculture workers from Mexico [43]. In a study of male adolescents in Spain, OP chlorpyrifos/chlorpyrifos-methyl exposure was negatively associated with estradiol levels [52]. In a cross-sectional study, estradiol was negatively associated with pyrethroid metabolites in the serum of 322 men with fertility problems living in Massachusetts [19]. In a study of 522 adolescents in Ecuador in the EPSINA study, urinary neonicotinoid acetamiprid was positively associated with 17β-estradiol, in boys only [26]. Atrazine, an herbicide, has been negatively associated with estradiol in rats [53]

Few studies have examined the effect of pesticide exposure, beyond organochlorines, on DHEA or cortisol levels [18, 23, 54, 55]. A study of 117 adolescent males from the Environment and Childhood (INMA)-Granada cohort in Spain found OP exposure was associated with an increase in DHEA levels [52]. While other classes of pesticides remain understudied and human studies are nascent, in a study of 143 male farmworkers in northern Thailand exposed to neonicotinoids (NNI), found that imidacloprid (a NNI) exposure was positively associated with DHEA and testosterone [23].

Little research has been done on pesticide exposure in children or adolescents who live in proximity to pesticide spray sites and endocrine disruption and few studies have incorporated geospatial constructs, studied floriculture or greenhouse agriculture, or included schools in the analysis, as children spend approximately one quarter to one third of the day at school.

The objective of this study was to evaluate the association of residential proximity to pesticide spray sites and greenhouse floriculture areas near households with concentrations of testosterone, estradiol, DHEA, and cortisol in adolescents in the agricultural county of Pedro Moncayo, Ecuador, as part of the Study of Secondary Exposures to Pesticides among Children, Adolescents and Adults (ESPINA, Exposición Secundaria a Plaguicidas en Niños, Adolescentes, y Adultos). Pedro Moncayo is an agricultural county with one of the highest concentrations of floricultural production in the Americas, and Ecuador is the second largest exporter of roses in the world [56]. Floricultural crops, roses specifically, are frequently grown in greenhouses and intensely sprayed with various pesticides such as insecticides (organophosphates, organochlorines, neonicotinoids, and pyrethroids) fungicides and to a lesser extent herbicides [57] in Pedro Moncayo. Previous findings within ESPINA suggested that home proximity to greenhouse floriculture crops within 275 m is associated with lower child acetylcholinesterase (AChE) activity, an enzyme that is inhibited by cholinesterase inhibitor insecticides, including organophosphates and carbamates [58]. We hypothesized, given previous research and trends in the literature, that increasing residential or school proximity to pesticide spray sites and larger crop areas near homes or schools (representing increased exposure) are associated with disruptions in hormones, altering levels of testosterone, estradiol, DHEA, and cortisol among adolescents. We also hypothesized that the presence of an agricultural or floricultural worker in the household is associated with hormone alteration in their children, considering that agricultural workers take home pesticides on their clothing [59].

## Methods

### Ethical review

Institutional review boards at the University of California San Diego (IRB #160,060), Universidad San Francisco de Quito (IRB #206-047E), and the Ministry of Public Health of Ecuador reviewed and approved this study. Both parents and participants were informed about the study, and informed consent was obtained from individuals ≥ 18 years old and parents of younger children. Permission of the participation of minor children was obtained from parents and child assent was also obtained from minors. As this is not a clinical trial, no clinical trial number is necessary.

### Research design

This research utilizes data obtained from the longitudinal prospective cohort study, ESPINA [60]. ESPINA aims to evaluate associations of background pesticide exposures in agricultural settings with short- and long-term alterations in the development of children and adolescents into adulthood. Study participants reside in the agricultural county of Pedro Moncayo, Ecuador, where approximately 21% of the adult population works in industrial floriculture, primarily growing roses in greenhouses for export [57].

#### Participant recruitment

**Fig. 1:**
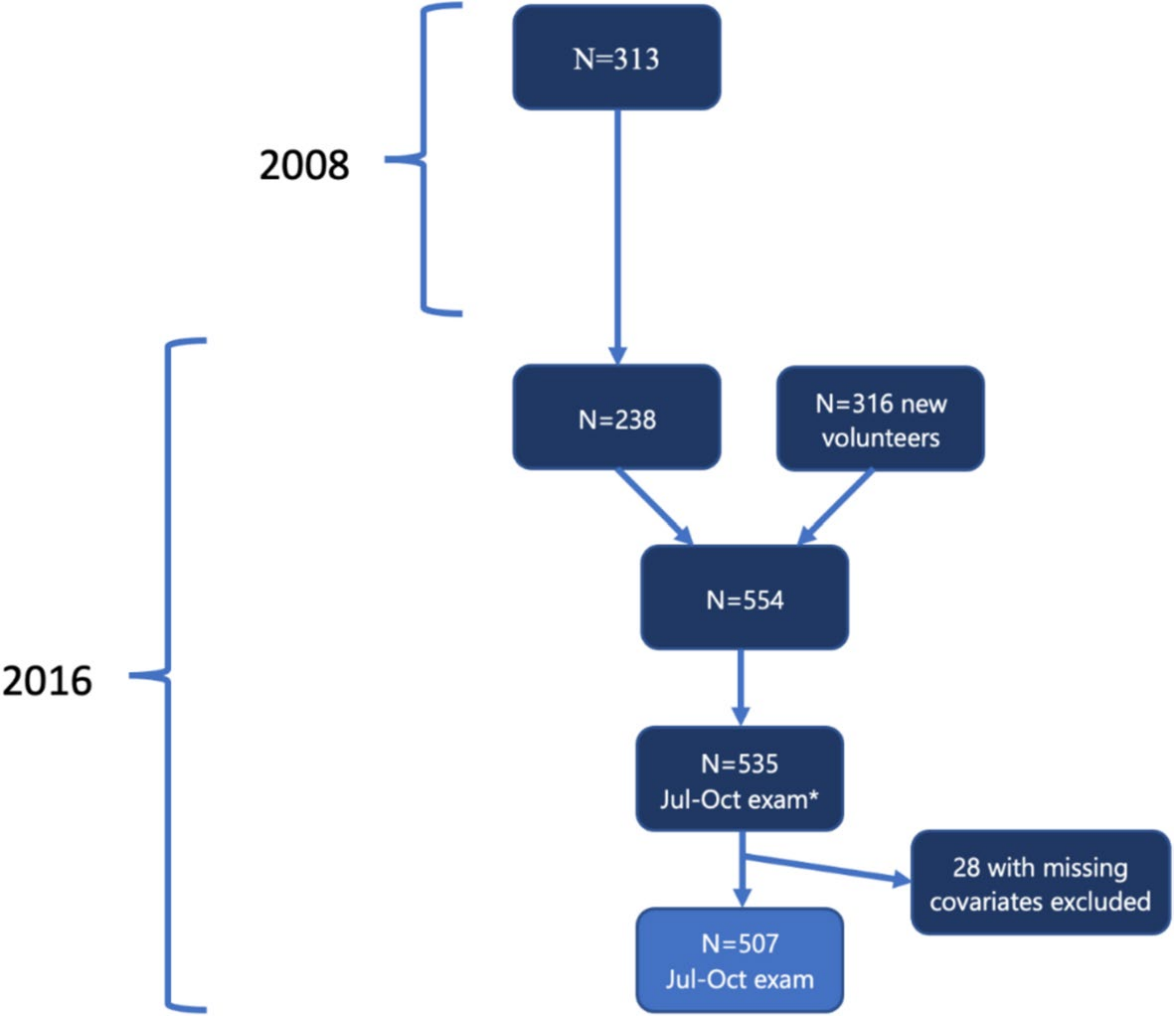
Participant flow chart

In 2008, 313 children (4–6 years of age) were recruited and examined, and the cohort was expanded in 2016 to 554 participants, which included 2 follow-up exams: April with *N* = 330 adolescent participants (12–17 years) and July–October with 535 participants recruited from the System of Local and Community Information (SILC). SILC, a database that contains geospatial information and health data, was used to recruit participants which included the 2004 Survey of Access and Demand of Health Services in Pedro Moncayo (SAHS-PM 2004). The present analyses include data from 507 participants from the July–October examination in 2016, who had all exposure, outcome, and covariates of interest (Fig. 1). Additional recruitment information has been described previously in [60] and [26].

#### Study site

**Fig. 2:**
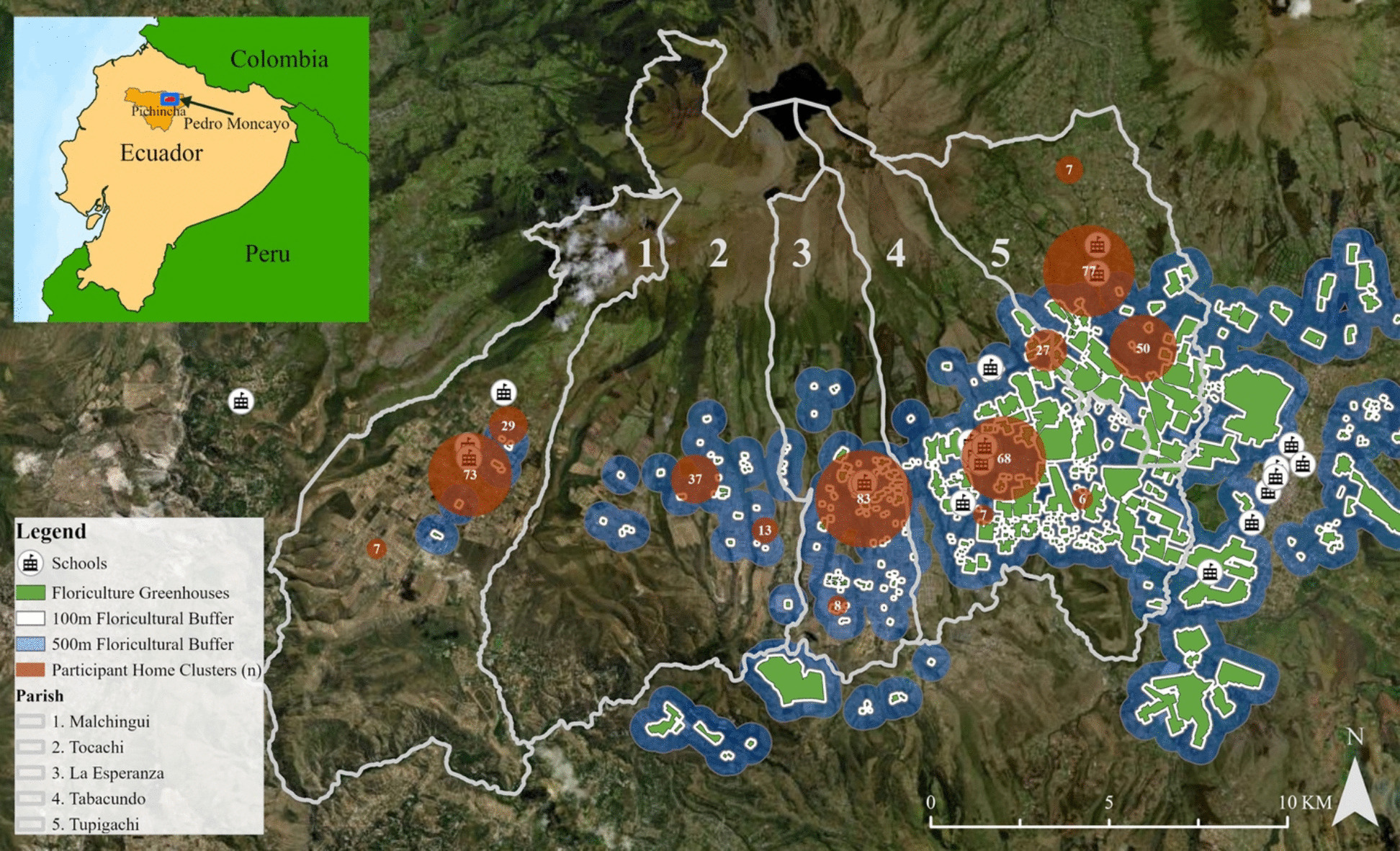
Study Region of Pedro Moncayo, Ecuador. The floriculture greenhouses are representative of 2016 data. Participant home clusters with less than 5 participants were masked. The ArcGIS World imagery basemap sources are Esri, DigitalGlobe, GeoEye, i-cubed, USDA FSA, USGS, AEX, Getmapping, Aerogrid, IGN, IGP, swisstopo, and the GIS User Community, and the World Topo Map with sources Esri, DeLorme, HERE, TomTom, Intermap, increment P Corp., GEBCO, USGS, FAO, NPS, NRCAN, GeoBase, IGN, Kadaster NL, Ordnance Survey, Esri Japan, METI, Esri China (Hong Kong), swisstopo, MapmyIndia, and the GIS User Community

Pedro Moncayo County has 5 parishes, and most floricultural crops are located in the two most eastern parishes of Tabacundo and Tupigachi. Participants living across all 5 parishes of Pedro Moncayo were included in ESPINA, providing considerable variability of exposure based on distance of homes to flower plantations. In 2016, greenhouse floricultural crop land comprised 4.47% of the geographic area of Pedro Moncayo (Fig. 2).

#### Data collection

In 2016, information on demographics, socioeconomic status, and history of pesticide exposure was gathered from parents in home interviews [57]. Participants were examined between July and October 2016 in schools of the county. Height was measured using a height board based on World Health Organization (WHO) guidelines, and weight was measured by a calibrated digital scale (Tanita model 0108 MC,Corporation of America, Arlington Heights, IL). WHO child growth standards were used to determine the body mass index (BMI)-for-age and height-for-age z-scores.

Sexual maturation rating (SMR) was assessed using Tanner Staging based on self-reported breast size and pubic hair growth/distribution for girls, and pubic hair growth/distribution for boys, using modified Tanner drawings from Rasmussen et al. as reference [61, 62]. Tanner staging, otherwise known as sexual maturity rating, serves as a guide to physical development and is used to identify secondary sex characteristics of puberty in adolescents [63].

#### Geospatial variables

**Fig. 3:**
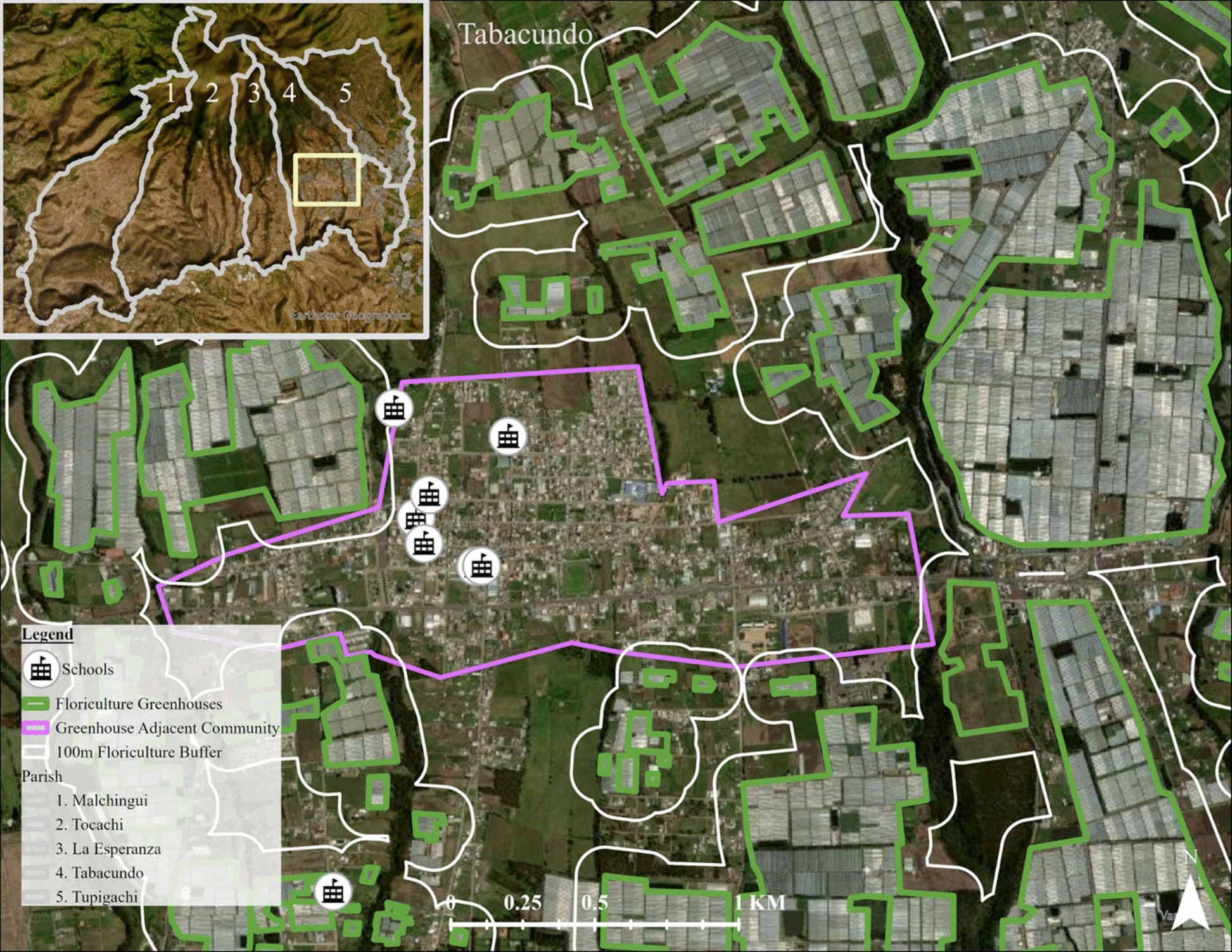
Map of a sub-section of the study region in Pedro Moncayo and the proximity of households to greenhouse floriculture. The image source is ArcGIS basecamp with an image of the region. The ArcGIS World imagery basemap sources are Esri, DigitalGlobe, GeoEye, i-cubed, USDA FSA, USGS, AEX, Getmapping, Aerogrid, IGN, IGP, swisstopo, and the GIS User Community

Geographical coordinates of homes and schools were obtained using global positioning system receivers and the perimeters of flower crops were manually determined from satellite imagery from the same time period. Schools were located using Google Maps. Homes’ distance to floricultural greenhouses and distance from participant’s school to the nearest greenhouse were calculated (Fig. 3). We calculated the total surface area (m^2^) of floricultural greenhouses within each buffer region to the home or school. The calculation of distance between the child’s home and the nearest flower crop, as well as the total area of flower crops within various buffers (100 m, 150 m, 200 m, 300 m, and 500 m), was calculated using ArcGis version 3.34.6. The projection 32,717 (WGS 84/UTM zone 17S). While distance is commonly used in the literature, surface area of agriculture within buffer zones around homes may provide a better measure of agricultural density within each radius [6, 64].

#### Salivary hormone measurements

Concentrations of 17β-estradiol, testosterone, cortisol, and DHEA were measured using enzymatic assays (Salimetrics, Carlsbad, CA) at the University of California San Diego in passive-drool samples. Participants were asked to collect samples upon awakening to control for circadian differences. Estradiol concentrations were only measured in boys, as the levels of estradiol in adolescent women vary according to the stage of their menstrual cycle and have within person variability.

### Statistical analysis

Covariates were identified a priori using subject expertise, and included gender, race, age, height-for-age z-score to adjust for nutritional status, monthly parental salary (household income), tanner sexual maturation score, awakening response (time of saliva sample collection – time of awakening) to adjust for diurnal variation in hormones [65], and cohabitation with an agricultural worker.

Participant characteristics are reported as mean (standard deviation) or medians (interquartile range [IQR]) for normally distributed and skewed continuous variables respectively and counts and proportions for categorical variables. These values are reported for four stratified categories of distance from home to the nearest floricultural crop (Table 1). A simple linear regression model was used to calculate the association (p-trend) between each variable with the distance to the nearest floricultural plantation.

**Table 1:** Participant characteristics across residential distance to the nearest floricultural crop, (*N* = 507)

N	**Categories of Residential Distance to the Nearest Floricultural Crop**					
	**Total**	** < 150 m**	**151–300 m**	**301–500 m**	** > 500 m**	**p-trend**
	507 (100%)	168 (33%)	80 (16%)	78 (15%)	181 (36%)	-
Gender						< 0.01
Female, %	260 (51%)	95 (37%)	40 (15%)	45 (17%)	80 (31%)	
Male, %	247 (49%)	73 (30%)	40 (16%)	33 (13%)	101 (41%)	
Race						< 0.001
Indigenous, %	112 (22%)	18 (16%)	26 (23%)	24 (21%)	44 (39%)	
Mestizo, %	395 (78%)	150 (38%)	54 (14%)	54 (14%)	137 (35%)	
Age, years	14.47 (1.75)	14.34 (1.77)	14.54 (1.82)	14.35 (1.87)	14.61 (1.66)	0.16
Height-for-age z-score	−1.48 (0.91)	−1.40 (0.92)	−1.51 (0.80)	−1.50 (0.88)	−1.54 (0.96)	0.26
Monthly Household Income, USD	585.39 (402.84)	614.48 (517.13)	560.70 (252.15)	621.81 (453.31)	553.61 (299.38)	0.34
Tanner score	2.90 (0.95)	2.85 (0.95)	2.96 (0.98)	2.84 (0.98)	2.95 (0.93)	0.21
Awakening response	0.50 (0.96)	0.52 (1.01)	0.58 (1.25)	0.43 (0.78)	0.48 0.84)	0.28
Cohabitation with floricultural worker						0.29
Yes, %	341 (67%)	104 (30%)	56 (16%)	61 (18%)	120 (35%)	0.29
No, %	166 (33%)	64 (39%)	24 (14%)	17 (10%)	61 (37%)	0.29
Cortisol (pg/ml)						
Female	0.20 (0.14, 0.30)	0.21 (0.15, 0.32)	0.21 (0.15, 0.32)	0.20 (0.14, 0.27)	0.19 (0.13, 0.30)	0.56
Male	0.21 (0.13, 0.31)	0.23 (0.14, 0.32)	0.26 (0.12, 0.27)	0.26 (0.14, 0.31)	0.19 (0.12, 0.31)	0.37
Testosterone (pg/ml)						
Female	32.06 (23.19, 43.09)	33.94 (25.86, 42.54)	38.66 (25.74, 47.17)	28.21 (21.89, 41.22)	29.23 (21.34, 40.23)	0.76
Male	58.94 (34.76, 99.78)	61.14 (35.40, 98.81)	55.24 (29.49, 93.48)	49.95 (34.84, 93.16)	60.86 (37.69, 108.09)	0.33
Estradiol (pg/ml)						
Female		-	-	-	-	-
Male	0.43 (0.30, 0.59)	0.43 (0.31, 0.62)	0.41 (0.25, 0.63)	0.41 (0.29, 0.54)	0.44 (0.32, 0.58)	0.27
DHEA (pg/ml)						
Female	81.45 (45.78, 133.01)	81.47 (45.83, 128.46)	90.99 (44.48, 176.83)	71.30 (32.72, 99.55)	81.83 (48.46, 127.30)	0.53
Male	43.77 (22.14, 78.52)	41.29 (19.98, 65.10)	39.82 (25.83, 76.18)	28.28 (14.25, 75.60)	51.09 (25.87, 94.08)	0.25

Hormone values presented are median (25 percentile, 75 percentile), continuous covariates are presented as mean (SD), and categorical covariates are presented as count (percentage)

*Abbreviations*: *USD* United States Dollars, *SD* Standard deviation

Multiple linear regression was used to study the relationship of residential distance to floricultural crops with 17β-estradiol, testosterone, cortisol, and DHEA while controlling for all covariates listed in Table 1. We also used multiple linear regression to study the association of floricultural crop areas near homes and hormones, using crop areas within 100 m, 150 m, 200 m, 300 m, and 500 m of homes as continuous variables. We used natural log-transformed values of areas of crops within buffers around homes, residential distance to the nearest crop, and hormone levels in all models, as these variables had a skewed distribution. Since the natural logarithm function was applied to both the independent variable (distance or area) and hormone concentration values, our regression model followed a log–log model. Therefore, linear regression coefficients were interpreted on a multiplicative scale for each variable, and the beta coefficients were transformed by the following equation:

$$\left(2^\beta\;-\;1\right)\cdot100\%$$

This equation reframed the beta coefficients as a percentage increase in hormone concentrations due to doubling the independent variable, whether it is distance to the nearest crop or crop areas within buffers around homes. Estimates of the percent difference in hormones after doubling distance or area variable with respect to the hormone variables were reported. Analysis included only participants with non-zero values.

We conducted gender-stratified analyses for all associations considering that most hormone concentrations measured are dramatically different between males and females. We also analyzed the association of the presence of an agricultural or floricultural worker in the household with hormone alteration, considering that agricultural workers take home pesticides on their clothing [59].

Distance-stratified linear regression analysis was conducted to assess whether associations varied between distance quantiles of subjects. First, the model was stratified by those who lived within or beyond 300 m from the nearest floricultural crop as it was approximately the median distance. Previous findings suggested that home proximity to greenhouse floricultures within 275 m is associated with lower child AChE activity, reflecting greater exposure to cholinesterase inhibitor insecticides [2, 3]. Models were then further stratified using four categories: < = 150 m, 151–300 m, 301–500 m and > 500 m.

An additional model was run for participants who reported the school they attended (*N* = 477). We explored school distance to the nearest floricultural crop as an exposure construct in our models for 477 children with school data, and floricultural crop area within 100 m, 200 m, 300 m, and 500 m of the school, as children can spend approximately one-third of their weekdays in school. This models adjusted for the covariates previously described.

To visualize the trends, we plotted adjusted least squares (LS) means of each hormone with area or distance, adjusted for all model covariates. To calculate the adjusted LS means, 270 ranks were used for cortisol, testosterone, and DHEA, and 150 ranks were used for estradiol, as this hormone was only measured in males. The purpose of using adjusted LS means for visualization is that they provide visualization of covariate-adjusted observations (ranks). We visualized the trend line using a locally estimated scatterplot smoothing (LOESS) curve based on the adjusted ranks with a smoothness level of 0.75.

All statistical analyses were conducted using R 4.1.1.

## Results

### Participant characteristics

Of the 507 participants examined in 2016, 51% were female and the mean age was 14.47 years. Twenty-two percent of participants identified as Indigenous, 78% as Mestizo. Table 1 shows participants’ characteristics across categories of distance. Most participants lived with an agricultural worker (67%) (Table 1). The children examined in our study were substantially shorter for their age when compared to the WHO Child Growth Standard with a mean height-for age z-score of −1.48 (SD = 0.91). The median (25th-75th percentile) residential proximity to crops within 300 m of homes were 312.6 m (112.3 m, 653.2 m). The median (25th-75th percentile) residential crop areas within 300 m of homes was 0m^2^ (0m^2^, 7325m^2^). Of participants, 49% lived within 300 m of greenhouse floricultural crops. Mestizo participants lived disproportionately closer to greenhouse floriculture crops than indigenous participants (*p <* 0.001).

### Areas of greenhouse floriculture crops near homes and hormone concentrations

**Fig. 4:**
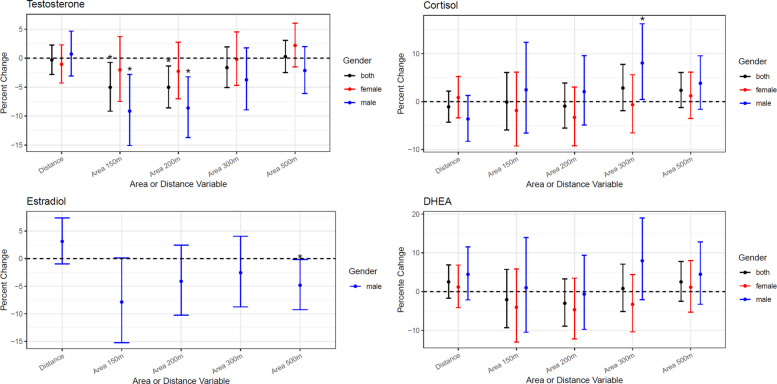
Percent difference in hormone concentration in adolescents after doubling residential distance to the nearest floricultural crop or crop areas within various buffers (100–500 m) around homes. All models are adjusted for gender, race, age, height-for-age z-score, monthly parental salary, tanner sexual maturation score, awakening response, and cohabitation with an agricultural worker. Asterisks (*) denote a p-value under 0.05. The points represent estimates while the error bars represent 95% confidence intervals

We found greater floriculture crop areas within 500 m of adolescents’ residences were associated with altered hormone concentrations (testosterone, estradiol, cortisol) in boys, but not girls. The associations for testosterone and estradiol were strongest for 150 m buffers, while the associations with cortisol were strongest for a 300 m buffer. *Testosterone.* We found that doubling crop areas within 150 m and 200 m buffers around homes were associated with reduced concentrations of testosterone (−5.03% (95% CI: −9.15%, −0.73%)) and (−5.03% (−8.58%, −1.34%)), respectively (Fig. 4 and Supplemental Table 1). The LOESS curves allow us to visualize the slope differences (Supplemental Fig. 1). Gender stratified analyses showed that these associations with reduced testosterone concentrations were only observed in boys (150 m buffer: −9.14% [95% CI: −15.08%, −2.79%]; 200 m buffer: −8.60% [95% CI: −13.69%, −3.20%]) and not girls (150 m buffer: −2.02% [95% CI: −7.48%, 3.76%]; 200 m buffer: −2.26% [95% CI: −7.03%, 2.77%]). We also found that the presence of an agricultural or floricultural worker in the household was associated with a decrease in testosterone (−10.87% (0.71%, 20.00%)). *Estradiol.* Doubling crop areas within 150 m (−7.87% (95% CI: −15.24%, 0.13%)) was borderline significant, while doubling crop areas within 500 m was significantly associated with lower estradiol, measured in boys only (−4.82% (95% CI: −9.26%, −0.17%)). Overall, the associations of crop areas with testosterone and estradiol weakened with wider buffer areas (Fig. 1). *Cortisol.* Doubling crop areas within 300 m was associated with greater cortisol concentration in boys (8.05% (95% CI: 0.45%, 16.23%)) but not girls (−0.65% (95% CI: −6.54%, 5.60%)). *DHEA.* No statistically significant associations were observed with DHEA, although stronger associations in boys vs girls were observed with crop areas within 300 m.

### Residential proximity to greenhouse floriculture crops and hormones

**Fig. 5:**
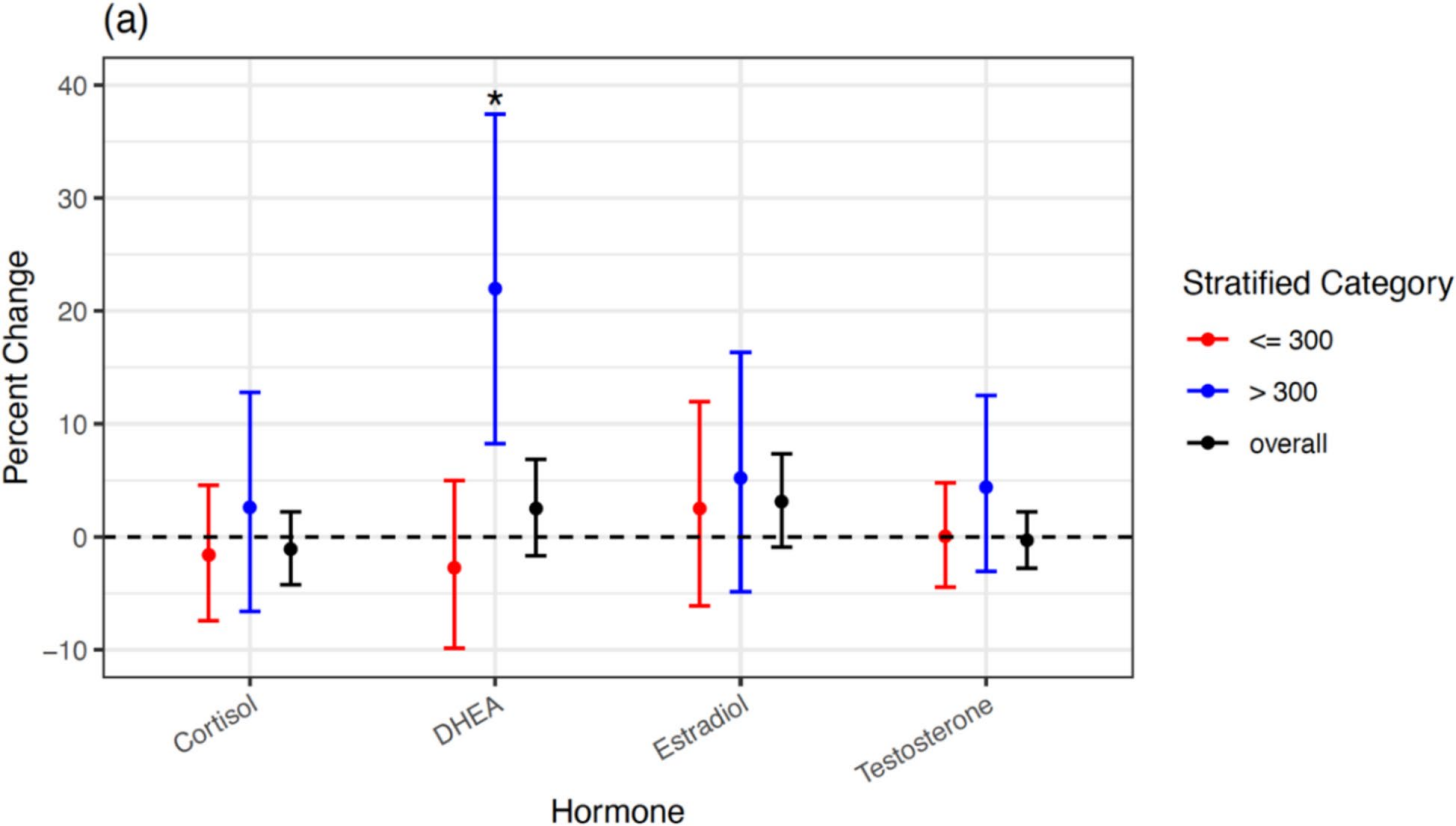
Percent difference in hormone concentration in adolescents after doubling residential distance to crops, stratified by distance greater and less than 300 m. All models are adjusted for gender, race, age, height-for-age z-score, monthly parental salary, tanner sexual maturation score, awakening response, and cohabitation with an agricultural worker. Asterisks (*) denote a p-value under 0.05. The points represent estimates while the error bars represent 95% confidence intervals

**Fig. 6:**
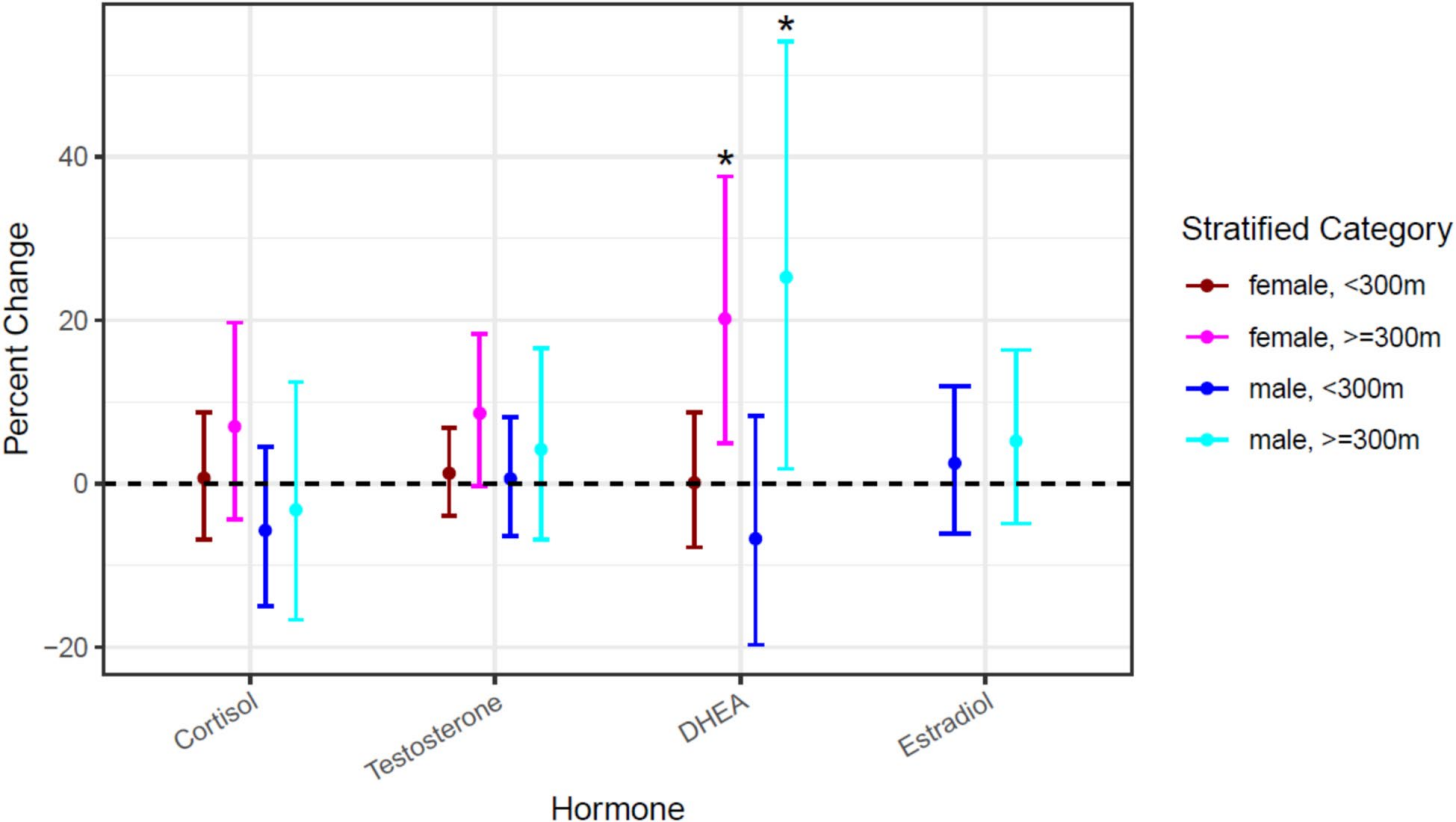
Percent difference in hormone concentrations in adolescents after doubling residential distance, stratified by gender and distance greater and less than 300 m. All models are adjusted for race, age, height-for-age z-score, monthly parental salary, tanner sexual maturation score, awakening response, and cohabitation with an agricultural worker. Asterisks (*) denote a p-value under 0.05. The points represent estimates while the error bars represent 95% confidence intervals

**Fig. 7:**
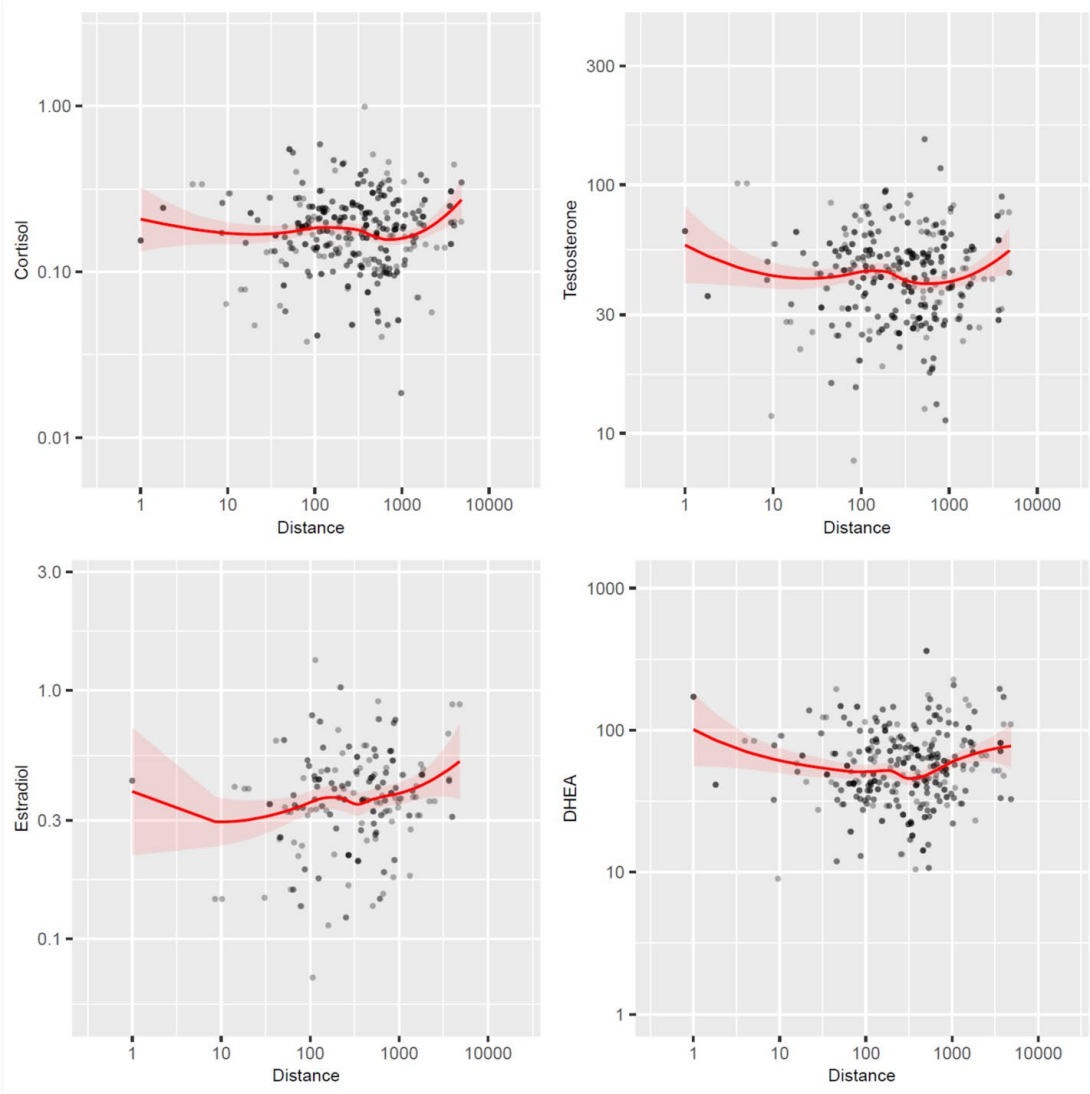
LOESS plots of the associations between residential distance to floricultural greenhouses and all four hormones. Points represent the adjusted least squares means of 270 ranks for cortisol, testosterone and DHEA, and of 150 ranks for estradiol. Adjusted covariates include gender, race, age, height-for-age z-score, monthly parental salary, tanner sexual maturation score, awakening response, and cohabitation with an agricultural worker. Both axes are on a logarithmic scale to reflect the transformations made in the model. The red line represents the LOESS curve of the relationship between distance and hormones, and pink areas around the lines are the 95% CI

In pooled and gender-stratified multivariate regression models, doubling residential proximity to the nearest greenhouse floriculture crops was not significantly associated with hormone levels, except for a borderline non-significant association with greater estradiol in boys (3.13% (95% CI: −0.93%, 7.36%)) (Fig. 4, Supplemental Table 1). In our distance-stratified analysis, we found that doubling the residential proximity to crops was associated with greater DHEA (21.98% (95% CI: 8.21%, 37.51%)) among participants living beyond 300 m; thus, greater distance from floriculture was associated with greater DHEA in boys by 25.2% (95% CI: 1.8%, 54.0%) and girls by 20.2% (95% CI: 5.0%, 37.6%) (Figs. 5 and 6, Supplemental Table 2). Slope differences can be visualized in the LOESS graphs (Fig. 7).

### School proximity to floricultural greenhouses and hormones

Our data included participant’s school proximity to crops for 477 individuals, and the number of participants with non-zero values of floricultural crop areas across various buffer sizes are shown in Supplementary Table 3. We found that doubling the crop area within 100 m of schools was associated with lower estradiol in boys (−6.70% (95% CI: −11.78%, −1.34%)) (Supplemental Table 4). No statistically significant associations were observed with other hormones.

## Discussion

This is the first study we know of to evaluate the associations of geospatial exposure constructs of pesticide drift, including residential and school distance to crops and crop areas near homes and schools, with some sex and adrenal hormones in children. We observed lower concentrations of testosterone in males with increasing greenhouse floriculture crop areas within either 150 m or 200 m of homes. Higher cortisol concentrations were also observed in boys with greater areas of crops within 300 m of homes. Higher cortisol has been associated with depression in boys [66, 67]. Greater crop area within 500 m was also significantly associated with lower estradiol, measured in boys. We also found that increasing distance (decreasing exposure) beyond 300 m from households from greenhouse floriculture was associated with significantly greater DHEA in boys and girls; DHEA is helpful in cognitive function and low levels are associated with depression [68]. We also included distance from school to nearest plantation and crop areas near schools, resulting in an additional significant association: doubling of area within 100 m of schools resulted in lower estradiol for boys.

Off-target pesticide drift from agricultural sites to nearby homes is an important source of pesticide exposure to agricultural populations. Studies have found that of the total pesticides sprayed in an area, up to 90% can be volatilized and/or drift with wind [1, 4, 8]. Pesticide drift has been shown to decay with distance, as the highest pesticide concentrations have been observed within 100 m of application sites,yet, concentrations can still be detected up to 4,100 m away from the center of the spray site in open field agriculture [8]. Evidence suggests that increased pesticide exposure occurs for those living in households within 500 m of agricultural crops, including in our ESPINA research in Pedro Moncayo, Ecuador and in the CHAMACOS study in California, USA [2, 3, 58, 64, 69]. Furthermore, our work in ESPINA found that cholinesterase activity was lower among children living withing 275 m of greenhouse floriculture crops [2, 3]. While greenhouse agriculture may have less drift potential than open field agriculture, our research in Ecuador suggests it still was significant enough to be associated with decreased cholinesterase, which regulates the nervous system and interacts closely with the endocrine system [2]. This research provides validation of residential proximity and area of greenhouse floriculture near homes and schools as indicators of chronic pesticide exposure. In our research, area of greenhouses within specific radii around children’s homes and schools was a better explanatory variable than distance, as it tends to be a better construct of drift and may better capture cumulative pesticide exposure.

We found lower levels of testosterone in male adolescent boys living when doubling crop areas with 150 and 200 m of homes. Lower levels of testosterone can lead to detachment of germ cells which can lead to lower sperm count and increased sperm abnormalities [35]. We also found lower estradiol in boys with a greater area of households within 500 m of greenhouses and with greater greenhouse area within 150 m of schools. Low levels of estradiol can affect musculoskeletal and bone development, reproductive function, and metabolism [70]. Within 300 m, greater area, was associated with higher cortisol in boys. Cortisol is often referred to as a stress hormone, and chronic elevation can lead to obesity, type two diabetes, cardiovascular disease and neurodegenerative disease [71, 72]. Increased distance away from greenhouse floriculture was associated with significantly greater DHEA in males and females; and DHEA is important for hormone production, and immune and cognitive function [68],suggesting that children raised more than 300 m from greenhouse floriculture, where there is less pesticide drift, may enhance DHEA production.

Our findings add to the body of literature on pesticide-related endocrine alterations. In animal and human studies, OP and organochlorine insecticide exposure have been linked to endocrine alteration. Animal studies point to a decrease in estradiol after exposure to OPs, chlorpyrifos and chlorpyrifos-methyl, specifically [50, 51], which concurs with our findings. In human studies, estradiol was negatively associated with pyrethroid metabolites in serum [19]. There are few studies conducted in adolescent populations that research hormone levels as they relate to pesticide exposure and/or proximity to pesticide spray sites. One study of male adolescents in Spain exposed to organophosphate pesticides (specifically chlorpyrifos/chlorpyrifos-methyl OPs), found reduced estradiol levels [52]. Similarly, we found that proximity to floricultural greenhouses was associated with lower estradiol for boys. We also found that increased area of floriculture within 100 m of household residence was associated with lower estradiol in boys. In a longitudinal study of floriculture workers in Mexico, exposure to OP pesticides was associated with decreased serum testosterone levels [43]. We found a similar relationship in urinary metabolites for testosterone in adolescent boys who grow up in proximity to floricultural crops.

In addition, residential distance to crops and crop areas close to children’s homes past studies have found that the number of farmworkers in the household can also increase exposure via take-home pathways, including pesticide residues on the skin, clothing, and equipment [73–75]. This was evident in our results,the presence of an agricultural worker in the household was associated with a decrease in testosterone by 10.87 (0.71, 20.00) percent.

This study has some limitations. We only collected one hormone sample per participant, despite potential circadian fluctuations throughout the day, which limits the assessment of intra-individual variability. We mitigated this by asking participants to collect saliva samples upon awakening and then by adjusting the statistical models by awakening response (time of saliva sample collection minus awakening time). Additionally, longitudinal analyses during various developmental stages would improve characterization and temporality of the association between pesticide exposure and hormone changes, as well as provide information of susceptible developmental periods. Future ESPINA data collections and related studies should incorporate designs that capture daily or cycle-phase–specific estradiol fluctuations among females to improve interpretability and support sex-specific comparisons. Although, distance to agricultural areas and crop areas near homes and schools are valid measures of exposure to pesticides, we did not consider wind patterns that could have influenced level of exposure which potentially introduced misclassification bias. Roses are grown year-round in greenhouses in Ecuador, as the rose plant has a long lifespan and Ecuador provides consistent, homogenous year-round temperatures at the equator. In Pedro Moncayo, roses are the primary crop, but other crops like corn, broad beans, and strawberries are cultivated in open-air fields in this region. Currently, there are no maps which indicate where other agriculture crops were present in 2016 in Pedro Moncayo; thus, we could not account for potential pesticide drift originating from non-floricultural agriculture. Ecuador lacks pesticide use reporting systems that are present in other countries, thus we are unable to weight our distance exposure proxies based on reported application quantities and pesticide classifications used [76]. We are also limited by the sample size in each distance and residential area category which reduces our statistical power and may limit our ability to detect small effects.

This study has many advantages and contributions. We add to the limited geospatial research on proximity to pesticide spray sites and hormone alteration, especially in adolescents. Geospatial proximity to pesticide spray sites may capture the cumulative effects of multiple pesticide exposures over time that measuring one pesticide at a single time fails to capture. Methodologically, in addition to distance, we also used crop area near homes within specific radii to better document pesticide exposure. The surface area of agriculture within buffer zones around homes provides a better measure of agricultural density within each radius whereas the distance from the home to the nearest agricultural plot reflects how close the child’s household is to agriculture. The two measures are related yet capture different levels of potential exposure; using both constructs can be a more accurate exposure proxy. The improved exposure characterization of the agricultural density variable may be why we observed more significant associations with that variable than with residential distance to crops. We also added school proximity data to our geospatial analysis. This is insightful because children spend roughly one-third of their weekdays in school. These covariates of school distance and area should be further explored in future exposure research, as has been suggested by those involved in the CHAMACOS Study [77].

## Conclusions

This is the first study to evaluate geospatial determinants of off-target pesticide drift from greenhouse floriculture to nearby homes, including residential and school proximity to crops and crop surface areas, in relation to hormone alterations. Geospatial proximity to pesticide spray sites may serve as a proxy for cumulative pesticide exposure in agricultural communities where residents are exposed to a diverse and large range of insecticides, herbicides, and fungicides. The substantial number of pesticides available globally and the temporal variability of their use pose significant challenges for comprehensive exposure assessment. We found greater greenhouse floriculture area near children’s homes and schools was linked to hormonal changes in boys. Specifically, we found these associations for boys: testosterone levels were lower with greater crop area within 150–200 m, cortisol levels were higher with more crop area within 300 m, and estradiol levels, important for musculoskeletal and bone development, were lower with more crop area within 500 m of homes. In addition, as children spend a significant portion of their day at school, we found that as greenhouse area increased within 100 m of a child’s school, estradiol levels were lower for boys, but not girls. We did find one association for boys and girls: DHEA, helpful in cognitive function, was significantly higher for boys and girls who lived greater than 300 m from greenhouse floriculture.

From a population health perspective, pesticide exposure can have a wide range of direct and indirect health effects on people who work in agriculture. Increasingly, we see the health risks for those who grow up in proximity to agricultural where pesticides are heavily sprayed. Unfortunately, despite growing evidence of such adverse effects, most countries have not implemented protective measures to monitor and reduce such exposures in residential or school areas that are in proximity to pesticide spray sites. Children who grow up near pesticide spray sites are especially vulnerable, as their bodies are developing, and their life is still before them. To efficiently monitor at-risk groups, including children and adolescents, further research is needed to better understand the impact of chronic pesticide exposure on the endocrine system, in adolescence and over the life-course. This study has provided data on this at-risk population with high exposure to pesticides that can inform future efforts to monitor and protect vulnerable populations living in agricultural settings. Moreover, the results could guide future policies regarding safe distances from pesticide spray areas for residential and school areas.

## Supplementary Information

Supplementary Material 1: Supplemental Table 1. Percent difference in hormone concentration after doubling residential distance to the nearest floricultural crop or crop areas within various buffers around homes. Supplemental Table 2. Distance-stratified percent difference in hormone concentration after doubling residential distance to the nearest floricultural crop. Supplemental Table 3. School distance to nearest greenhouse and greenhouse floriculture crop area within various buffers and Hormone Sample Sizes*. Supplemental Table 4. School Proximity to Floricultural Greenhouses and hormone concentration in adolescents. Supplemental Figure 1. Adolescent Household Distance and Area from Pesticide Spray Sites and Testosterone levels. 

## Data Availability

The data that support the findings of this study are not openly available due to reasons of sensitivity and are available from the corresponding author upon reasonable request.
