# Supplementary material for: Sex and adrenal hormone alteration in Ecuadorian adolescents with home and school proximity to floriculture crop area
Source: Environ Health. 2026 Apr 25;25:51. doi: 10.1186/s12940-026-01291-x (PMC13248469; doi:10.1186/s12940-026-01291-x)
Supplement: Supplementary file 1 — Supplementary Material 1: Supplemental Table 1. Percent difference in hormone concentration after doubling residential distance to the nearest floricultural crop or crop areas within various buffers around homes. Supplemental Table 2. Distance-stratified percent difference in hormone concentration after doubling residential distance to the nearest floricultural crop. Supplemental Table 3. School distance to nearest greenhouse and greenhouse floriculture crop area within various buffers and Hormone Sample Sizes*. Supplemental Table 4. School Proximity to Floricultural Greenhouses and hormone concentration in adolescents. Supplemental Figure 1. Adolescent Household Distance and Area from Pesticide Spray Sites and Testosterone levels. [file 12940_2026_1291_MOESM1_ESM.docx]

**Sex and Adrenal Hormone Alteration in Ecuadorian Adolescents with Home and School Proximity to Floriculture Crop Area**

**Supplemental Materials**

**Supplemental Table 1:** Percent difference in hormone concentration after doubling residential distance to the nearest floricultural crop or crop areas within various buffers around homes

| Independent Variable | Percent of Participants | Cortisol  (N = 507) | Testosterone  (N = 504) | DHEA  (N = 488) | Estradiol  (N = 241) |
| --- | --- | --- | --- | --- | --- |
| Distance | 100 |  |  |  |  |
| Both |  | -1.09 (-4.28, 2.21) | -0.30 (-2.80, 2.26) | 2.51 (-1.70, 6.90) | - |
| Male |  | -3.63 (-8.29, 1.28) | 0.72 (-3.09, 4.68) | 4.46 (-2.14, 11.52) | 3.13 (-0.95, 7.38) |
| Female |  | 0.84 (-3.40, 5.26) | 1.05 (-4.28, 2.30) | 1.20 (-4.14, 6.84) | - |
| Area within 150m | 33 |  |  |  |  |
| Both |  | - 0.10 (-5.93, 6.09) | -5.03 (-9.15, -0.73) ^*^ | -2.08 (-9.31, 5.72) | - |
| Male |  | 2.46 (-6.57, 12.37) | -9.14 (-15.08, -2.79)^*^ | 1.00 (-10.48, 13.97) | -7.87 (-15.24, 0.13) |
| Female |  | -1.87 (-9.26, 6.13) | -2.02 (-7.48, 3.76) | -4.03 (-13.00, 5.87) | - |
| Area within 200m | 39 |  |  |  |  |
| Both |  | -0.95 (-5.55, 3.86) | -5.03 (-8.58, -1.34)^*^ | -3.01 (-8.90, 3.26) | - |
| Male |  | 2.08 (-4.90, 9.58) | -8.60 (-13.69, -3.20)^*^ | -0.64 (-9.76, 9.39) | -4.11 (-10.25, 2.44) |
| Female |  | -3.30 (-9.25, 3.04) | -2.26 (-7.03, 2.77) | -4.68 (-12.19, 3.48) | - |
| Area within 300m | 49 |  |  |  |  |
| Both |  | 2.83 (-1.91, 7.79) | -1.63 (-5.08, 1.94) | 0.80 (-5.12, 7.08) | - |
| Male |  | 8.05 (0.45, 16.23)^*^ | -3.73 (-8.93, 1.78) | 7.95 (-2.09, 19.03) | -2.55 (-8.74, 4.07) |
| Female |  | -0.65 (-6.54, 5.60) | -0.18 (-4.69, 4.54) | -3.29 (-10.39, 4.38) | - |
| Area within 500m | 64 |  |  |  |  |
| Both |  | 2.35 (-1.27, 6.10) | 0.26 (-2.48, 3.09) | 2.51 (-2.50, 7.79) | - |
| Male |  | 3.83 (-1.60, 9.55) | -2.13 (-6.09, 1.99) | 4.48 (-3.27, 12.85) | -4.82 (-9.26, -0.17) ^*^ |
| Female |  | 1.20 (-3.54, 6.17) | 2.20 (-1.51, 6.04) | 1.13 (-5.29, 7.99) | - |

* pvalue < 0.05; estimates represent percentage changes derived from log–log regression models and correspond to a doubling of the exposure variable

**Supplemental Table 2:** Distance-stratified percent difference in hormone concentration after doubling residential distance to the nearest floricultural crop

| Stratified Distances | Percent of Participants | Cortisol (N = 507) | Testosterone (N = 504) | Estradiol (N = 241) | DHEA (N = 488) |
| --- | --- | --- | --- | --- | --- |
| <= 300m | 49 | -1.60 (-7.45, 4.61) | 0.05 (-4.48, 4.79) | 2.51 (-6.24, 12.08) | -2.74 (-9.94, 5.05) |
| > 300m | 51 | 2.61 (-6.67, 12.82) | 4.41 (-3.12, 12.52) | 5.22 (-4.94, 16.47) | 21.98 (8.21, 37.51)^*^ |

* pvalue < 0.05

| Stratified Distances | Percent of participants | Cortisol (N = 507) | Testosterone (N = 504) | Estradiol (N = 241) | DHEA (N = 488) |
| --- | --- | --- | --- | --- | --- |
| <= 150m | 32 | 0.00 (-7.01, 7.54) | 0.20 (-5.10, 5.80) | 6.03 (-5.38, 18.81) | -5.92 (-14.24, 3.21) |
| 151-300m | 16 | -31.93 (-64.24, 29.58) | -17.17 (-49.58, 36.08) | -11.71 (-53.51, 67.65) | -33.60 (-70.41, 49.00) |
| 301-500m | 15 | -16.64 (-62.88, 87.18) | -38.96 (-69.02, 20.25) | -8.85 (-65.88, 143.47) | 15.44 (-71.34, 364.98) |
| > 500m | 37 | 18.77 (3.43, 36.37) ^*^ | 10.94 (-0.43, 23.60) | 6.98 (-7.14, 23.24) | 8.73 (-6.70, 26.71) |

* pvalue < 0.05; analysis included only participants with non-zero values; estimates represent percentage changes derived from log–log regression models and correspond to a doubling of the exposure variable

**Supplemental Table 3: School Distance and Hormone Sample Sizes***

| Independent Variable | Cortisol | Testosterone | Estradiol | DHEA |
| --- | --- | --- | --- | --- |
| Distance | 477 | 474 | 224 | 460 |
| Area within 100m | 104 | 104 | 43 | 101 |
| Area within 200m | 185 | 184 | 82 | 182 |
| Area within 300m | 231 | 229 | 105 | 226 |
| Area within 500m | 300 | 298 | 132 | 293 |

*Sample sizes are presented with non-zero values


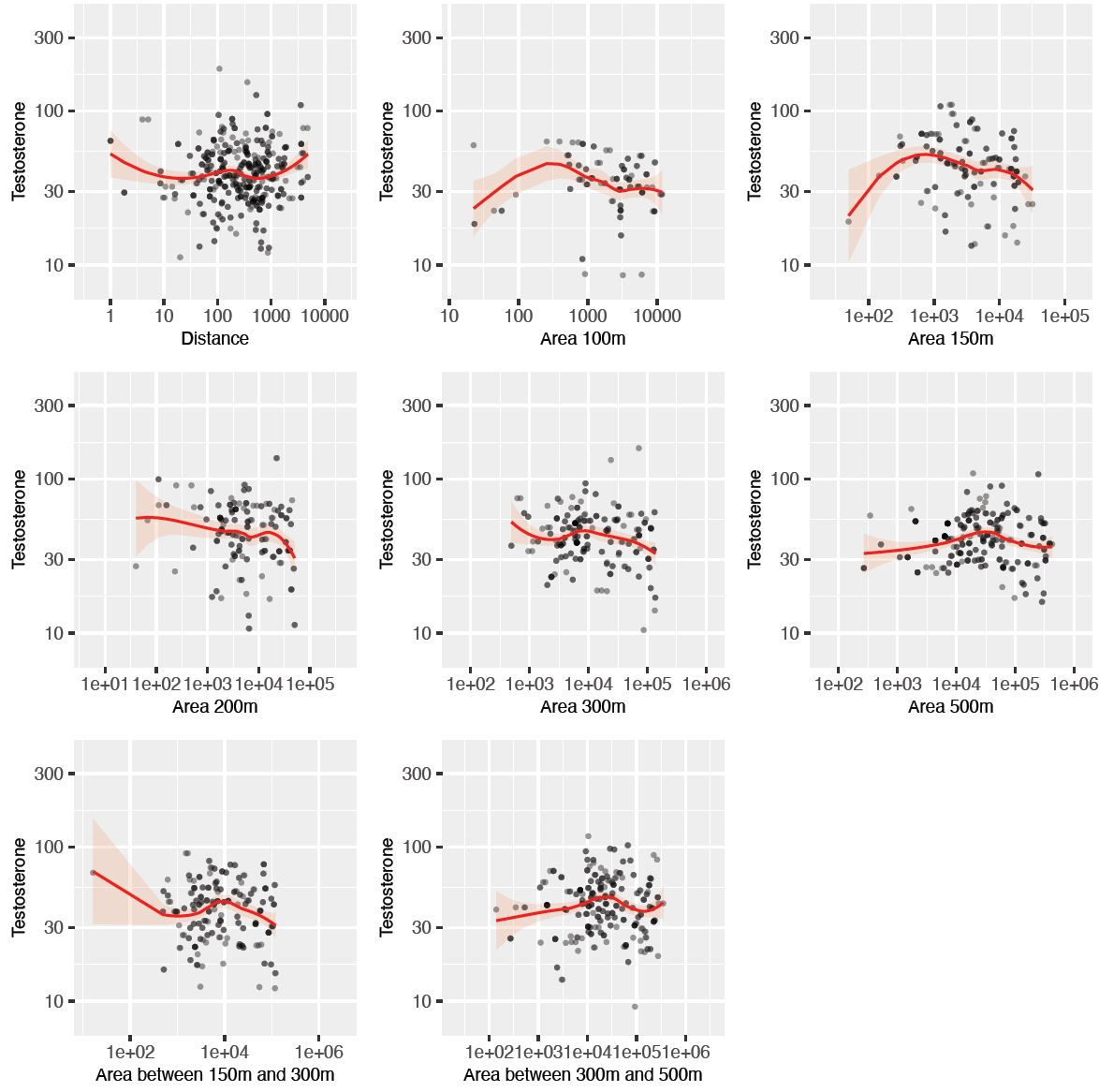


**Supplemental Figure 1:** Adolescent Household Distance and Area from Pesticide Spray Sites and Testosterone levels

**Supplemental Table 4:** School Proximity to Floricultural Greenhouses and hormone concentration in adolescents:

| **Doubling school distance to crops or crop areas near schools and percent difference (95%CI) in hormone concentration** | | | | | |
| --- | --- | --- | --- | --- | --- |
| **Independent Variable** | **Gender** | **Cortisol** | **Testosterone** | **Estradiol** | **DHEA** |
| Distance | Both | -0.25 (-4.53, 4.23) | -1.87 (-5.07, 1.44) | NA | 3.54 (-1.71, 9.07) |
|  | Male | -1.31 (-7.52, 5.31) | -2.45 (-7.12, 2.45) | -0.77 (-5.83, 4.58) | 2.97 (-4.60, 11.15) |
|  | Female | 0.83 (-4.99, 7.00) | -1.48 (-5.80, 3.04) | NA | 3.78 (-3.23, 11.30) |
| Area within 100m | Both | -1.38 (-4.91, 2.28) | 1.81 (-1.11, 4.81) | NA | -1.52 (-6.68, 3.92) |
|  | Male | -4.34 (-15.36, 8.11) | 0.90 (-8.57, 11.35) | -6.70 (-11.78, -1.34)^*^ | -9.10 (-23.78, 8.40) |
|  | Female | 0.26 (-4.32, 5.06) | 2.35 (-1.43, 6.28) | NA | 3.34 (-3.38, 10.53) |
| Area within 200m | Both | 0.11 (-1.76, 2.23) | 0.35 (-1.20, 1.92) | NA | 0.27 (-2.32, 2.92) |
|  | Male | -0.60 (-7.30, 6.59) | -1.04 (-6.19, 4.39) | -1.39 (-4.11, 1.42) | -1.27 (-9.79, 8.06) |
|  | Female | 0.68 (-1.73, 3.14) | 1.13 (-0.73, 3.02) | NA | 1.04 (-2.08, 4.25) |
| Area within 300m | Both | -0.18 (-1.90, 1.58) | 0.60 (-0.69, 1.91) | NA | -1.19 (-3.33, 0.99) |
|  | Male | -0.12 (-6.43, 6.63) | -0.56 (-5.24, 4.36) | 0.58 (-1.80, 3.01) | -2.70 (-10.22, 5.46) |
|  | Female | -0.22 (-2.32, 1.93) | 1.29 (-0.29, 2.90) | NA | -0.34 (-2.92, 2.32) |
| Area within 500m | Both | 0.51 (-0.72, 1.76) | 0.26 (-0.68, 1.21) | NA | -0.32 (-1.89, 1.28) |
|  | Male | 0.98 (-3.93, 6.15) | 0.62 (-3.11, 4.50) | 0.54 (-1.05, 2.16) | -0.08 (-6.21, 6.46) |
|  | Female | 0.16 (-1.46, 1.81) | -0.03 (-1.25, 1.22) | NA | -0.48 (-2.52, 1.60) |

* p-value < 0.05; analysis including only participants with non-zero values; estimates represent percentage changes derived from log–log regression models and correspond to a doubling of the exposure variable
